# Expanded gram-negative antimicrobial prophylaxis reduces surgical site infection in orthopedic surgery

**DOI:** 10.1017/ash.2026.10845

**Published:** 2026-09-09

**Authors:** Scott C. Roberts, Marjorie Golden, Edwin Ruiz Fuentes, Nicole Colandrea, Suzanne Dahlberg, Lee E. Rubin, Richard A. Martinello

**Affiliations:** 1 Yale School of Medicine, New Haven, USA; 2 Yale School of Public Health, USA; 3 Yale New Haven Hospital, USA

## Abstract

Gentamicin was added to the standard of care antimicrobial prophylaxis in 5,500 patients receiving orthopedic surgery. Patients who received gentamicin had lower surgical site infection rates (2.1% vs 3.7%, *P* = .020). This was most pronounced in patients with hip fracture who underwent repair (0.6% vs 2.9%, *P* = .007).

## Introduction

Surgical site infections (SSIs) occur in approximately 2%–2.5% of patients who undergo orthopedic surgery.^1,2^ Appropriate perioperative antimicrobial prophylaxis represents a core practice in SSI reduction. Antibiotic prophylaxis effectiveness largely depends on drug selection, dosage, and timing. According to joint clinical practice guidelines, a minimum of 2 g of cefazolin should be administered to adult patients within 60 minutes before surgical incision for orthopedic surgeries.^3^

In some centers where pathogens other than staphylococci or streptococci are likely, additional agents active against those pathogens could be considered.^3^ One such agent with expanded gram-negative activity is gentamicin, an aminoglycoside which has activity against pathogens such as *Pseudomonas aeruginosa* in addition to gram-positives including *Staphylococcus aureus*.^3^ However, data in clinical practice are conflicting and may depend on procedure type. One center determined that addition of gentamicin to cefazolin reduced the incidence of SSI in patients undergoing hip arthroplasty, while another center found no difference in patients who underwent knee arthroplasty.^4,5^ Given an anecdotal increase in SSIs due to gram negative pathogens at our center, gentamicin was added as an adjunctive antimicrobial for prophylaxis in patients undergoing orthopedic surgery.

## Methods

A retrospective cohort study evaluating patients who underwent orthopedic surgery at Yale New Haven Hospital, a 1,541 bed tertiary care hospital, from January 1, 2019, through July 31, 2021, was performed. Procedures included hip arthroplasty, knee arthroplasty, spine fusion or laminectomy, and hip fracture with repair through either arthroplasty, intramedullary nailing, or open reduction and internal fixation. SSI was defined according to the National Health and Safety Network (NHSN) definitions and determined through surveillance by the infection prevention team.

The standard of care perioperative prophylaxis for orthopedic surgery at our institution was a weight-based cefazolin, with vancomycin used if patients screened positive for methicillin-resistant *Staphylococcus aureus* (MRSA). Starting in July 2017, a single dose of gentamicin at 2 mg/kg was recommended to be given within 60 minutes of incision in addition to the routine standard of care prophylaxis, however administration was per surgeon discretion. This “expanded gram-negative antibiotic prophylaxis” (EGNAP) protocol was implemented after internal review noted a high proportion of SSIs were due to gram-negative organisms. Only patients with surgical wounds classified as clean were included. For statistical analysis, the surgeries were divided into three groups: routine hip and knee arthroplasty, spine fusion and laminectomy, and hip fracture with repair, which was further subdivided into arthroplasty versus non-arthroplasty. SSI rates according to receipt of gentamicin were adjusted for American Society of Anesthesiologists (ASA) score with logistic regression. SPSS version 28.0 was used, and methods included Fisher’s Exact test and χ^2^. This quality improvement initiative was exempt from Yale University Institutional Review Board (IRB) review.

## Results

Between January 1, 2019 to July 31, 2021, a total of 5,500 patients underwent the prespecified orthopedic surgical procedures, including 2,534 (46.1%) who underwent hip or knee arthroplasty, 2,067 (37.6%) who underwent spine fusion or laminectomy, and 899 (16.3%) who sustained hip fracture with surgical repair, of which 275 (30.6%) received hip arthroplasty and 624 (69.4%) received repair such as intramedullary nailing or open reduction and internal fixation (Tables 1–3). 4,882 of the 5,500 cases (88.8%) received the institutionally recommended gentamicin antimicrobial prophylaxis, while 618 (11.2%) did not.

**Table 1.** Demographics: hip + knee arthroplasty Table 1 long description.

Variable	No gentamicin (n = 65)	Gentamicin (n = 2,469)	*P*-value
Age (years)	64.6 (±12.3)	63.7 (±11.3)	.505
Sex			.715
Female	41 (63.1%)	1502 (60.8%)	
Male	24 (36.9%)	967 (39.2%)	
BMI	32.8 (± 8.2)	32.1 (±7.4)	.459
ASA score			.006
ASA1	0 (0.0%)	39 (1.6%)	
ASA2	17 (26.2%)	987 (40.0%)	
ASA3	45 (69.2%)	1,403 (56.8%)	
ASA4	3 (4.6%)	40 (1.6%)	
Procedure duration (minutes)	100.9 (±53.2)	84.5 (±37.0)	.016

**Table 2.** Demographics: spine fusion and laminectomy Table 2 long description.

Variable	No gentamicin (n = 276)	Gentamicin (n = 1791)	*P*-value
Age (years)	59.1 (±16.2)	60.0 (±14.4)	.401
Sex			.293
Female	121 (43.8%)	846 (47.2%)	
Male	155 (56.2%)	945 (52.8%)	
BMI	30.4 (±6.7)	30.1 (± 6.5)	.563
ASA score			.361
ASA1	11 (4.0%)	61 (3.4%)	
ASA2	87 (31.5%)	620 (34.6%)	
ASA3	166 (60.1%)	1,056 (59.0%)	
ASA4	12 (4.3%)	54 (3.0%)	
Procedure duration (minutes)	121.8 (±73.1)	170.0 (±104.9)	<.001

**Table 3.** Demographics: hip fracture with repair Table 3 long description.

Variable	No gentamicin (n = 277)	Gentamicin (n = 622)	*P*-value
Age (years)	80.8 (±13.3)	82.6 (±10.8)	.042
Sex			.115
Female	205 (74.0%)	428 (68.8%)	
Male	72 (26.0%)	194 (31.2%)	
BMI	24.4 (±5.2)	24.5 (±5.1)	.933
ASA score			.484
ASA1	3 (1.1%)	2 (0.3%)	
ASA2	45 (16.2%)	108 (17.4%)	
ASA3	190 (68.6%)	441 (70.9%)	
ASA4	39 (14.1%)	70 (11.3%)	
ASA5	0 (0.0%)	1 (0.2%)	
Procedure duration (minutes)	54.3 (±35.6)	52.8 (±30.7)	.549

When adjusting for ASA score, patients who received gentamicin compared to those who did not have lower SSI rates across all procedures (2.1% vs 3.7%, *P* = .020). This difference was most pronounced in the hip fracture with repair group (0.6% vs 2.9%, *P* = .007). There were lower rates of SSI in hip and knee arthroplasty (2.2% vs 6.2%, *P* = .059) and spine fusion and laminectomy (2.4% vs 4.0%, *P* = .131) but these were not statistically significant (Tables 4–6).

**Table 4.** SSI rate according to appropriate gentamicin use across all surgeries Table 4 long description.

	No gentamicin (n = 618)	Gentamicin (n = 4,882)	*P*-value
Hip and knee arthroplasty (n = 2,534)	4/65 (6.2%)	54/2,469 (2.2%)	.059
Spine fusion and laminectomy (n = 2,067)	11/276 (4.0%)	43/1791 (2.4%)	.131
Hip fracture with repair (n = 899)	8/277 (2.9%)	4/622 (0.6%)	.007
All cases (n = 5,500)	23/618 (3.7%)	101/4,882 (2.1%)	.020

**Table 5.** SSI rate in hip fracture with intramedullary nailing and open reduction with internal fixation Table 5 long description.

	No gentamicin (n = 253)	Gentamicin (n = 371)	*P*-value
No SSI (n = 616)	247 (97.6%)	369 (99.5%)	.047
SSI (n = 8)	6 (2.4%)	2 (0.5%)	

**Table 6.** SSI rate in hip fracture with arthroplasty Table 6 long description.

	No gentamicin (n = 24)	Gentamicin (n = 251)	*P*-value
No SSI (n = 271)	22 (91.7%)	249 (99.2%)	.003
SSI (n = 4)	2 (8.3%)	2 (0.8%)	

Within the hip fracture and repair group, the difference was greatest in the hip fracture with arthroplasty group (0.8% vs 8.3%, *P* = .003), although there was a lower SSI rate in those with repair as well (0.5% vs 2.4%, *P* = .047) (Tables 5 and 6). Twelve patients who underwent hip fracture with arthroplasty developed an SSI. Eight (66.7%) were culture negative and 4 (33.3%) were culture positive. Of the four with positive cultures, three (75%) did not receive gentamicin, but had gentamicin susceptible organisms grow: one with *Staphylococcus aureus*, one with *Enterobacter cloaecae*, and one that was polymicrobial with *Enterococcus faecium* and *Enterobacter cloaecae*.

Comparing patients who received gentamicin versus those who did not receive gentamicin, there was no difference in postoperative acute kidney injury (0.3% vs 0.6%, *P* = .175) nor *C. difficile* infection (0.2% vs 1.0%, *P* < .001).

## Discussion

Routine addition of gentamicin to the standard of care antimicrobial prophylaxis was associated with a decrease SSI rate in patients with hip fractures who underwent repair at a single center. A statistically significant difference was not seen across other orthopedic procedures, including routine hip arthroplasty, knee arthroplasty, and spine fusion and laminectomy, although all groups trended toward lower SSI rates. The benefits from gentamicin are likely explained by both its expanded gram-negative coverage and anti-staphylococcal properties,^6^ organisms which accounted for most of our culture-proven SSIs in patients who underwent hip arthroplasty with repair. The closer proximity of surgical sites to endogenous sources of contamination, such as the groin, may contribute to a higher risk of exposure to gram-negative flora.^7^ This may be compounded in the hip fracture population, who may have increased age, medical comorbidities, and immobility that could predispose to greater frequencies of bowel and urinary flora contamination.

Gentamicin use appeared to be safe; there was no difference in *C. difficile* infection or acute kidney injury. However, we did not evaluate other potential adverse events such as ototoxicity or downstream antimicrobial resistant pathogens. At least one other study found a single high dose of gentamicin was safe perioperatively with no increase in nephrotoxicity.^8^ It is possible our low rates *C. difficile* infection may be explained by a shorter hospital length of stay after orthopedic surgery procedures. It is also possible there was confounding by indication, as clinicians may have been less keen to prescribe gentamicin in patients at higher risk for adverse events.

These findings are partially consistent with previous studies. One group found no difference in SSI rates for knee or hip fracture with arthroplasties between patients receiving gentamicin versus standard antibiotic prophylaxis.^4,9^ Another study also found no benefit in patients undergoing total knee arthroplasty, but a benefit was observed in patients who underwent routine hip arthroplasty.^5^ In that center, adjunctive gentamicin was instituted after observing that 30% of SSIs were caused by gram-negative bacilli. It may be necessary to assess the types of pathogens that are causing SSIs in a procedure-specific manner when adopting strategies for antibiotic prophylaxis, consistent with guideline recommendations.^3^ This may have been effective for our institution given the higher gram-negative SSI rate when the addition of gentamicin was recommended, and it is unclear if this could be generalized to other centers without a high incidence of gram-negative SSIs. Local surveillance systems could be used to inform perioperative antimicrobial prophylaxis strategies.

Limitations of this study include the single center retrospective nature. There may have been selection bias in surgeons who preferentially administered gentamicin, and adjustment was with ASA score alone.

## Table 1. Long description

A table comparing demographics and surgical procedure details between patients who received gentamicin and those who did not. The table has 8 rows and 5 columns. The columns are labeled Variable, No gentamicin (n = 65), Gentamicin (n = 2,469), and P-value. The rows are labeled Age (years), Sex, BMI, ASA score, and Procedure duration (minutes). Each row provides specific data for patients without gentamicin and with gentamicin, along with the corresponding P-value. For example, the Age (years) row shows an average age of 64.6 for patients without gentamicin and 63.7 for patients with gentamicin, with a P-value of 0.505. The Sex row breaks down the number of female and male patients in each group. The BMI row shows the average BMI for each group. The ASA score row provides the distribution of ASA scores among patients. The Procedure duration (minutes) row shows the average procedure duration for each group.

Navigate back to Table 1..

## Table 2. Long description

A table comparing demographics of spine fusion and laminectomy patients with and without gentamicin. The table has 7 rows and 4 columns. Column headers are Variable, No gentamicin (n = 276), Gentamicin (n = 1791), and P-value. Row 1: Age (years), 59.1 (16.2), 60.0 (14.4), .401. Row 2: Sex,, , .293. Row 3: Female, 121 (43.8%), 846 (47.2%), . Row 4: Male, 155 (56.2%), 945 (52.8%), . Row 5: BMI, 30.4 (6.7), 30.1 (6.5), .563. Row 6: ASA score,, , .361. Row 7: ASA1, 11 (4.0%), 61 (3.4%), . Row 8: ASA2, 87 (31.5%), 620 (34.6%), . Row 9: ASA3, 166 (60.1%), 1,056 (59.0%), . Row 10: ASA4, 12 (4.3%), 54 (3.0%), . Row 11: Procedure duration (minutes), 121.8 (73.1), 170.0 (104.9), .001.

Navigate back to Table 2..

## Table 3. Long description

The table compares demographics and surgical details of patients who received gentamicin and those who did not. It has 10 rows and 5 columns. The columns are labeled Variable, No gentamicin (n = 277), Gentamicin (n = 622), and P-value. The rows are labeled Age (years), Sex, Female, Male, BMI, ASA score, ASA1, ASA2, ASA3, ASA4, ASA5, and Procedure duration (minutes). The values in the table are as follows: Age (years): No gentamicin 80.8 (±13.3), Gentamicin 82.6 (±10.8), P-value .042; Sex: No gentamicin 205 (74.0%), 72 (26.0%), Gentamicin 428 (68.8%), 194 (31.2%), P-value .115; BMI: No gentamicin 24.4 (±5.2), Gentamicin 24.5 (±5.1), P-value .933; ASA score: No gentamicin 3 (1.1%), 45 (16.2%), 190 (68.6%), 39 (14.1%), 0 (0.0%), Gentamicin 2 (0.3%), 108 (17.4%), 441 (70.9%), 70 (11.3%), 1 (0.2%), P-value .484; Procedure duration (minutes): No gentamicin 54.3 (±35.6), Gentamicin 52.8 (±30.7), P-value .549.

Navigate back to Table 3..

## Table 4. Long description

A table comparing surgical site infection rates with and without gentamicin use across different surgeries. The table has four rows and three columns. Column headers are No gentamicin (n = 618), Gentamicin (n = 4,882), and P-value. Row labels are Hip and knee arthroplasty (n = 2,534), Spine fusion and laminectomy (n = 2,067), Hip fracture with repair (n = 899), and All cases (n = 5,500). Row 1: 46/56 (6.2 percent), 54/2,469 (2.2 percent), 059. Row 2: 11/276 (4.0 percent), 43/1,791 (2.4 percent), 131. Row 3: 8/277 (2.9 percent), 4/622 (0.6 percent), 007. Row 4: 23/618 (3.7 percent), 101/4,882 (2.1 percent), 020.

Navigate back to Table 4..

## Table 5. Long description

A table comparing SSI rates in hip fracture treatments with and without gentamicin. The table has two columns for treatment groups: No gentamicin (n = 253) and Gentamicin (n = 371). It also includes a P-value column. The rows are labeled No SSI (n = 616) and SSI (n = 8). For the No SSI row, 247 (97.6 percent) patients had no SSI without gentamicin, and 369 (99.5 percent) had no SSI with gentamicin. The P-value is .047. For the SSI row, 6 (2.4 percent) patients had SSI without gentamicin, and 2 (0.5 percent) had SSI with gentamicin.

Navigate back to Table 5..

## Table 6. Long description

The table presents data on surgical site infection (SSI) rates in patients who received gentamicin compared to those who did not. It has four rows and three columns. The columns are labeled ‘No gentamicin (n = 24)’, ‘Gentamicin (n = 251)’, and ‘P-value’. The rows are labeled ‘No SSI (n = 271)’, ‘SSI (n = 4)’, and include the respective percentages and counts. Row 1: No SSI, No gentamicin, 22 (91.7 percent); Gentamicin, 249 (99.2 percent); P-value, .003. Row 2: SSI, No gentamicin, 2 (8.3 percent); Gentamicin, 2 (0.8 percent); P-value, .003.

Navigate back to Table 6..
